# Improving outcome of sensorimotor functions after traumatic spinal cord injury

**DOI:** 10.12688/f1000research.8129.1

**Published:** 2016-05-27

**Authors:** Volker Dietz

**Affiliations:** 1 Spinal Cord Injury Center, Balgrist University Hospital, Forchstrasse 340, Zürich, CH-8008, Switzerland

**Keywords:** Spinal cord injury, trauma, treatment, spinal cord repair, neuroprotective, neuroregenerative

## Abstract

In the rehabilitation of a patient suffering a spinal cord injury (SCI), the exploitation of neuroplasticity is well established. It can be facilitated through the training of functional movements with technical assistance as needed and can improve outcome after an SCI. The success of such training in individuals with incomplete SCI critically depends on the presence of physiological proprioceptive input to the spinal cord leading to meaningful muscle activations during movement performances. Some actual preclinical approaches to restore function by compensating for the loss of descending input to spinal networks following complete/incomplete SCI are critically discussed in this report. Electrical and pharmacological stimulation of spinal neural networks is still in the experimental stage, and despite promising repair studies in animal models, translations to humans up to now have not been convincing. It is possible that a combination of techniques targeting the promotion of axonal regeneration is necessary to advance the restoration of function. In the future, refinement of animal models according to clinical conditions and requirements may contribute to greater translational success.

## Introduction

Over the last few years, several approaches to improving the outcome of sensorimotor functions after spinal cord injury (SCI) have been established. These concern approaches to treating an acute traumatic SCI, exploiting neuroplasticity, and repairing damaged spinal cord by neuroprotective and neuroregenerative substances. For the latter approaches, a great number of animal studies have shown promising potential to translate the repair of the damaged spinal cord tracts to human SCI. In contrast, little progress has been made to treat impairment of the autonomic system, which impedes quality of life more than the ability of walking ^1^. Nevertheless, this short opinion paper is limited in its scope, i.e. it focuses on a few actual approaches to restoring sensorimotor functions after an SCI and intends to point out some of the problems in translating preclinical animal work to the clinic. In addition, it is thought to be selective and complementary to another review, ‘Recent advances in managing a spinal cord injury secondary to trauma’, provided by Fehlings’ group ^2^. The latter review discusses in more detail new approaches that are intended to be introduced into the clinic. Furthermore, a broader review about actual approaches to improving the outcome of sensorimotor functions after an SCI was recently published elsewhere ^3^.

## Management of acute injury

The actual treatment for SCI is focused on early decompression of the injured spinal cord, leading to an improved outcome ^4^. This approach is convincing and well established. The limitations are twofold: life-threatening complications can have a higher priority, and after an accident patients are frequently not directly transferred to a hospital that can provide adequate spine surgery. The consequences are inevitable delays. Nevertheless, there are clear indications to perform decompression surgery, e.g. by expansion duroplasty to improve circulation in the damaged spinal cord ^5^. Furthermore, by the application of new techniques of neuromonitoring from the injury site to guide the management of blood pressure, for example, and/or the preservation of spinal cord perfusion pressure at the injury site ^6^, additional damage might be avoided.

## Rehabilitation: exploitation of neuroplasticity

Great advances in the last few decades to exploit neuroplasticity, as well as the associated development of neurorehabilitation technology, as an important part of neurorehabilitation have become well established ^3^. Actual training approaches are directed to the activation of specific receptors necessary to lead to physiological limb activation ^7^ required for an improved outcome of function. Functional training programs based on animal experiments ^8^ dominate this part of rehabilitation. Furthermore, a spontaneously occurring neural re-organisation takes place for the compensation of paresis, such as the development of spastic muscle tone ^9^. This can be facilitated and adjusted by functional training.

During the last 20 years, functional training programs have led to the rapid development of neurorehabilitation technology (for review, see 10). Today, the training of functional movements of upper and lower limbs becomes assisted by an increasing number of robotic devices in combination with virtual reality programs, which facilitate training, provide feedback information, and allow longer training times (for review, see 11).

## Spinal cord repair: neuroprotection and neuroregeneration

The ultimate goal in treating SCI and improving function would in any case be spinal cord repair. A large number of neuroprotective and neuroregenerative agents exist and frequently show positive effects in animal models of SCI. However, the actual proven effects of these approaches are rather disappointing. Pilot studies and Cochrane reviews ^12^ indicate rather small beneficial effects of such agents (e.g. methylprednisolone ^13^). For example, an increase of four motor score points means functionally almost nothing, especially if considered with respect to possible side effects of such treatment (e.g. respiration problems and infections). Today, none of these substances are applied routinely in SCI patients (at least in Europe); early administration of methylprednisolone is established only in young patients suffering from an isolated fracture of the spine.

A number of current approaches to induce some regeneration of the spinal cord are on the way to becoming translated from the animal model to human beings. This is, indeed, an exciting area of research, as such approaches might lead to spinal cord repair. Unfortunately, the clinical significance of these approaches is frequently not critically questioned. Most of the agents have been studied in animal, usually rodent, models for more than 20 years and showed promising results. However, until now they have not yet been translated to humans or were not convincing in their effect ^3^. A promising approach to inducing regeneration was the application of olfactory ensheathing cells (OECs). In a carefully conducted study, such autologous cells were transplanted to chronic, motor complete SCI patients without any beneficial effect ^14^. What are the reasons for the problems in translating effects obtained in animal SCI models to the human condition? Some of them are listed below:

- In the rodent model, the spinal cord is usually transected, while in humans after a trauma the spinal cord is damaged over two to three segments; this impedes sufficient meaningful regeneration

- Several approaches were applied in complete human SCI (e.g. Nogo-Ab); however, in these patients, the remaining tissue bridges might not be sufficient to allow regeneration of tract fibres

- In animals, agents are usually applied directly after spinal cord transection; in the human condition, this is usually not feasible earlier than 2 to 3 weeks after trauma (phase 1 Nogo-Ab study in our center) due to, e.g., surgery, complications such as infections of the urinary tract or pneumonia, and patient consent

- Bridging was performed in chronic complete SCI patients (OEC transplantation); however, after 1 year, a neuronal dysfunction develops below the level of lesion that makes a success unlikely ^15, 16^

- Cervical SCI patients would most benefit from some regeneration for their quality of life; however, in contrast to thoracic lesions, a cervical SCI is associated with damage (up to 40% of paresis ^17^) of the peripheral nervous system (motoneurons and roots), and this makes the beneficial effects of regenerative substances and cells more unlikely

## Conclusions

In the future, most probably, only by a combination of neuroprotective and neuroregenerative strategies can real progress in spinal cord repair be achieved. As a consequence, it should be pointed out that any substantial progress in managing SCI critically depends on a close cooperation among clinicians, engineers, and basic scientists.
